# A Novel Case of Allergic Contact Dermatitis From *Gaultheria procumbens* Essential Oil in a Herbal Medical Ointment

**DOI:** 10.1111/cod.70185

**Published:** 2026-06-09

**Authors:** Florence Pasche Koo, Pierre Piletta‐Zanin, Sandrine Quenan, Olivier Aerts, Caroline Huber, Conrad Hauser

**Affiliations:** ^1^ Department of Dermatology Geneva University Hospital Geneva Switzerland; ^2^ Department of Dermatology University Hospital Antwerp and University of Antwerp Antwerp Belgium

**Keywords:** allergic contact dermatitis, case report, *Gaultheria procumbens* essential oil, herbal remedies

The use of herbal remedies has increased considerably over the last decade. Such remedies, containing plant derivatives or extracts, appeal greatly to many patients and are sometimes even preferred over standard medical therapies. Although patients are often convinced that these products bear no risk of provoking allergies, both allergic and irritant contact dermatitis have been attributed to them. We report a second case of severe allergic contact dermatitis from a herbal medical ointment containing *Gaultheria procumbens* essential oil [1].

## 1 Case Report

A 31‐year‐old nonatopic male patient had started to apply a compounded herbal ointment on a sprained ankle prescribed by a phytotherapist. 2 days later, an acute eczematous skin reaction appeared on the application site, as well as on the palm used to apply the ointment, and in the flexures of both forearms and knees. History revealed that about 3 years ago he had used another yet similar herbal ointment to treat a sore knee, at that time also complicated by the appearance of a localised dermatitis. Patch tests were performed with a baseline series (Hermal Chemie, Reinbek, Germany), and with the herbal ointment (‘as is’), mounted on a Finn chamber and occluded for 2 days. Patch tests showed an extremely positive reaction (+++) to the ointment on day (D)2 and 4. When testing with the individual raw ingredients (*Eucalyptus citriodora, Betula Allegohaniensis, Juniperus communis var Montana, Gaultheria procumbens, Gaultheria fragrantissima*), kindly provided by the therapist, positive reactions were only observed to *Gaultheria procumbens* essential oil (‘as is,’ semi‐open) (+++) and also when diluted 1% pet. on patch (++). Five control patients showed no reactions to the latter test preparation. Other positive patch tests (D4) were observed to: colophonium (+++), *Myroxylon pereirae* resin (++), fragrance‐Mix I (++) and lanolin (++).

Additional testing with 1%, 2% and 5% methylsalicylate, salicylaldehyde and benzylsalicylate (the former in olive oil and the latter two in petrolatum) was negative. As verification of the herbal ointment from 3 years ago showed the presence of *Gaultheria procumbens* , we diagnosed sensitization from this particular essential oil, with subsequent elicitation following renewed exposure.

## 2 Discussion

*Gaultheria procumbens* belongs to the Ericacea family. It is a tiny evergreen shrub with spicy aromatic edible berries, also called ‘Teaberry’ or ‘Wintergreen’ because it is an evergreen that continues photosynthesis through winter. Its leaves can be used to brew tea, or they can be distilled to obtain an oil. As the leaves contain methyl salicylate, the plant has been used in North‐American traditional medicine to alleviate symptoms from muscle and joint discomfort, various aches and pains [2]. Exposure to the plant may be a hazard for aspirin‐sensitive patients [3]. Our patient never experienced any particular reaction when taking aspirin. Moreover, patch tests with various salicylates, including methyl salicylate which has been postulated to be the responsible contact sensitizer [1], remained negative.

The concomitant reactions to colophonium, *Myroxylon pereirae* resin, or fragrance‐mix I are not surprising as many patients sensitised to essential oils co‐react to fragrances or related molecules. We cannot exclude that co‐sensitizations to these particular allergens occurred through the previous use of other topical remedies or cosmetics. Allergic contact dermatitis from essential oils in herbal remedies has been previously reported [4, 5, 6], sometimes in association with aromatherapy [7, 8], and they can also be found in cosmetics.

In conclusion, we report another case of acute allergic contact dermatitis from *Gaultheria procumbens* essential oil in a medical herbal ointment, yet the actual responsible sensitizer still remains unknown. As essential oils are very popular nowadays [9, 10], and as this is the second report concerning this particular oil, it is likely that new cases might occur in the near future.

## Author Contributions

**Caroline Huber:** validation. **Conrad Hauser:** writing – review and editing, methodology, validation. **Pierre Piletta‐Zanin:** methodology, investigation. **Sandrine Quenan:** validation. **Florence Pasche Koo:** writing – original draft, conceptualization, methodology, investigation, validation, supervision, project administration, writing – review and editing. **Olivier Aerts:** validation, methodology, writing – review and editing, conceptualization.

## Funding

The authors have nothing to report.

## Conflicts of Interest

The authors declare no conflicts of interest.

## Data Availability

The data that support the findings of this study are available on request from the corresponding author. The data are not publicly available due to privacy or ethical restrictions.
